# Positive Psychology Micro-Coaching Intervention: Effects on Psychological Capital and Goal-Related Self-Efficacy

**DOI:** 10.3389/fpsyg.2021.566293

**Published:** 2021-02-11

**Authors:** Alina Corbu, María Josefina Peláez Zuberbühler, Marisa Salanova

**Affiliations:** WANT Research Team, Department of Social Psychology, Universitat Jaume I, Castellón de la Plana, Spain

**Keywords:** positive psychology coaching, goal-related self-efficacy, psychological capital, goal attainment, short-term coaching, control trial, strengths-based intervention

## Abstract

Positive Psychological Coaching is receiving increasing attention within the organizational field because of its potential benefits for employees’ development and well-being (Passmore and Oades, 2014). The main aim of this study was to test the impact of a Positive Psychological Micro-Coaching program on non-executive workers’ psychological capital, and analyze how goal-related self-efficacy predicts goal attainment during the coaching process. Following a control trial design, 60 non-executive employees (35 in the experimental group and 25 in the waiting-list control group) from an automotive industry company participated in a Positive Psychological Micro-Coaching program over a period of 5 weeks. The intervention was grounded in the strengths-based approach and focused on setting a specific goal for personal and professional growth. The program consisted of a group session, three individual coaching sessions, and individual inter-session monitoring. Pre, post, and 4-month follow up measurements were taken to assess the impact on the study variables. Our results reveal that psychological capital increased significantly at post and follow-up times compared to baseline levels. In addition, results confirmed that goal-related self-efficacy predicted goal attainment during the micro-coaching process. Practical implications suggest that short-term positive psychological coaching is a valuable method for developing personal resources, such as psychological capital and to facilitate the goal achievement in non-executive employees, in order to reach work-related goals.

## Introduction

More than ever, organizations must deal with a highly competitive environment where changes occur at an overwhelming speed, transforming the way they work and function, and requiring employees to learn new skills and expertise in order to execute their task effectively. Accepting negative situations, such as unpredictable environment or emotional complexity of human nature, can lead to the development of different strategies for dealing with them (Wong, 2020). In order to achieve success, organizational change has become a necessity. The efficacy of any change effort relies on the employees’ attitude and readiness for change (Madsen et al., 2005). Therefore, it is important to empower employees by increasing their personal resources (i.e., self-efficacy) (Emsza et al., 2016) in order to prepare them to deal with organizational changes. There are different mechanisms used by individuals to handle challenging circumstances, such as techniques that help to control thoughts, emotions, and behaviors (i.e., coping strategies) before, during and after difficulties (Skinner and Zimmer-Gembeck, 2007). Emerging research demonstrates that coaching is a valuable tool for organizations during turbulence because it helps to improve skills and attitudes for suitable change management and achieve work demands and goals (Kombarakaran et al., 2008; Grant, 2014; Bickerich et al., 2018). More recently, the increasing recognition that well-being plays a significant role in organizational performance has resulted in coaching becoming more holistic and focusing more on the health and well-being of employees (Green and Palmer, 2018).

As Walsh et al. (2018) reported, happy people tend to be more successful in different areas of life. One’s happiness takes place not only when confronting the negative existential anxieties, but also as a result of focusing on the positive and developing positive resources (Wong, 2016). Therefore, higher levels of well-being-related positive psychological resources, such as optimism, self-efficacy, resilience, and hope, increase the probability of successfully facing organizational challenges. Taken together, these positive psychological resources build psychological capital (PsyCap), a psychological construct described as a state rather than a trait (Luthans and Youssef-Morgan, 2017) that can be developed, modified, and learned. Positive psychological coaching is an ideal methodology for building this positive construct because it offers an environment and characteristics that facilitate the learning process (Petersen, 2015). Similarly, goal-related self-efficacy (Grant and Greene, 2004). and goal attainment (Green and Spence, 2014) are other crucial factors that contribute to organizational success and can be worked on via positive psychological coaching (Palmer and Whybrow, 2005).

Accelerated advances at the legislative, technological, cultural, and economic levels have also influenced the automotive market. Addressing new challenges, such as product diversification, competition, and customer expectations requires higher levels of efficiency and resilience (Ivanov et al., 2018). Organizational flexibility has become a competitive advantage, and its development is related to the employees’ ability to adjust to a volatile environment, which in turn determines the organization’s success (Mendes and Machado, 2015). Organizations should focus on the employees’ personal resources to achieve excellent organizational results (Van Wingerden et al., 2017). Therefore, coaching is suggested as a successful solution to promote resource development and, hence, reach high performance levels (Bodein et al., 2013).

Although there is research on the impact of executive coaching on well-being-related abilities and goal achievement in organizational environments (Grant, 2013a, 2014, 2017), empirical studies that investigate these variables in non-executive employees or workplace coaching are still limited. In this study, we use workplace coaching as a more comprehensive concept that integrates coaching provided to all levels of employees (specifically non-executive employees) in a work environment, in order to improve work performance and job-related skills (Grant, 2013a). Therefore, scientific studies on the impact of positive psychological coaching, and particularly strengths-based coaching, on personal resources of non-executive employees (Green and Spence, 2014; Peláez et al., 2020) make an important contribution to the literature. Considering that positive psychological coaching aims to seek solutions rather than focusing on problems (Biswas-Diener, 2010; Green and Palmer, 2018), main indicators of its effectiveness are goal attainment (Grant et al., 2009, 2010; Minzlaff, 2019) and specific self-efficacy to accomplish goals (Moen and Allgood, 2009; de Haan et al., 2016). Thus, studying the influence of goal-related self-efficacy represents a step forward in further understanding the role of personal resources in the effectiveness of the coaching process.

To address this gap, a controlled design study is presented in order to provide scientific evidence about the effect of a Positive Psychological Micro-Coaching (PPMC; i.e., short-term and strengths-based) intervention on the PsyCap of non-executive workers and the relationship between goal attainment and goal-related self-efficacy. Our proposal is based on previous research indicating the effectiveness of PPMC in improving personal resources, well-being and performance, and that the coaching process is effective even with fewer sessions (i.e., micro-coaching) (Theeboom et al., 2013; Peláez et al., 2020).

## Theory and Hypotheses

### Positive Psychological Micro-Coaching

In the last decade, research on Positive Psychology arises to provide an evidence-based knowledge of human flourishing by studying the optimal functioning of people and organizations, focusing on their strengths, and positive characteristics (Salanova et al., 2019). Based on its existential-humanistic roots, Positive Psychology broadens its definition by integrating both negative and positive aspects of the human condition in order to grow and flourish (Wong, 2016). The way to cultivate positive emotions, cognitions, and behaviors is through positive psychology interventions (PPI). These interventions are designed to enhance: (1) positive aspects, (2) person-activity adjustment, (3) abilities of the individuals involved, and (4) the mechanisms of positive activities aimed at improving well-being (Lyubomirsky and Layous, 2013). The purpose of this approach, unlike traditional psychology, is to focus on positive experiences, factors and scenarios (Parks and Biswas-Diener, 2013). Previous research (Lomas and Ivtzan, 2016; Wong, 2020) argued that this point of view ignore the balance between positive and negative experiences, and suggest that “the most promising strategy to accomplish the mission of positive psychology is to confront the dark side of human existence and understand the unique experience and expression of well-being” (Wong, 2020, p. 3). In view of the world’s uncertainty and challenge, handling and overcoming life’s adversities is necessary to strengthen, and, even positively transform one’s personal resources.

An approach to strengths developed by Linley (2008) suggests that strengths consist on the ability to think, feel, and behave in ways that allow full and optimal functioning in the pursuit of desirable and valuable results (Linley and Harrington, 2006). In the workplace environment, employees who make a deliberate effort to apply strengths on their daily work are more productive, successful, and happy (Miglianico et al., 2020).

Recently, an applied sub-discipline of psychology named Coaching Psychology has emerged and can be understood as a learning process tailored to the coachees’ specific needs that strengthens their natural capacity for growth (Gallwey, 2014). A collaborative (Spence and Grant, 2007; Green and Spence, 2014), reflective, and goal-centered relationship is required to accomplish the desired outcomes (Smither, 2011). In order to optimize time and costs, the short-term coaching process could be a useful intervention for the organizations as the society change in a fast-paced, constant, and unpredictable way. Micro-coaching attempt to create an ambiance where the goal is specific and viable to achieve in a short-term. The main differences between a standard coaching process and micro-coaching resides in the definition of a specific and short-term feasible goal and in fewer number of sessions in micro-coaching (Peláez et al., 2020).

Build on the definitions of these terms, previous research suggested the integration of positive psychology and psychological coaching because both approaches focus on developing optimal functioning and utilizing individuals’ strengths for improvement (Linley and Harrington, 2005; Green, 2014). Based on this approach, the concept of positive psychological coaching emerges as a technique that uses positive psychology principles to provide a “positive diagnosis” (Biswas-Diener, 2009). Positive psychology applied to coaching allows the coachee to be conscious of his personal resources, and provides the conditions for the development of skills and abilities beyond the usual or prescribed professional roles (Castiello D’Antonio, 2018). van Zyl et al. (2020) propose a definition of positive psychological coaching based on positive psychological evidence-based approaches that describes a collaborative relationship between coach and coachee focused on discovering, cultivating, and applying personal resources to enhance positive states and facilitate personal/professional growth. In general, coaching has always focused on strengths because of their explicit use as tools for personal development (Biswas-Diener, 2010). Burke (2018) suggests that the use of strengths in the PPMC, and particularly in strengths-based coaching, is a key element in finding solutions to help coachees achieve their goals. Additionally, the assessment of character strengths benefits the coaching process by creating awareness, increasing confidence, and developing personal resources to improve performance (Burke and Passmore, 2019). Positive psychological coaching is a powerful methodology because it promotes positive psychological interactions, helps employees to develop positive psychological resources, and increases productivity (Biswas-Diener, 2010).

Some interventions indicate that the use of personal resources for personal and professional success is an efficient organizational strategy to promote beneficial outcomes. For example, Meyers and van Woerkom (2017) observed that a brief strengths intervention increased employees’ positive affect and PsyCap by identifying and developing strengths and their use in the work context.

In recent years, research in the field of psychological coaching has experienced significant growth at the level of organizational research and practice. Several meta-analyses and studies highlight the effectiveness of coaching (Theeboom et al., 2013; Lai and McDowall, 2014; Sonesh et al., 2015; Jones et al., 2016; Bozer and Jones; 2018). Currently, a growing number of professionals are using positive intervention strategies because they are linked to increased psychological resources, such as self-efficacy (Proctor et al., 2011) and the achievement of personal and organizational goals (Linley et al., 2010).

Furthermore, although there is empirical evidence about the influence of executive coaching on work-related outcomes, such as leadership skills (MacKie, 2014), findings on the effects of coaching on non-executive workers are still limited (Grant, 2013a). However, recent research has focused on applying the strengths-based coaching methodology to non-executive positions, analyzing the effectiveness of strength-based coaching in promoting well-being (i.e., work engagement) and job performance (Peláez et al., 2020). Nevertheless, more studies with controlled and longitudinal designs are needed to broaden and build on the effects of PPMC on work-related outcomes, such as PsyCap, and the role of self-efficacy in achieving goals during the process, considering the key role of these variables in a coaching process. In order to respond to these requests, this study aims to contribute to the research on the impact of a PPMC program on PsyCap and the relationship between self-efficacy and goal attainment in the coaching process.

### Positive Psychological Micro-Coaching and Psychological Capital

Luthans et al. (2007b) define PsyCap as

An individual’s positive psychological state of development, characterized by: (1) having confidence (self-efficacy) to take on and put in the necessary effort to succeed at challenging tasks; (2) making a positive attribution (optimism) about succeeding now and in the future; (3) persevering toward goals and, when necessary, redirecting paths to goals (hope) in order to succeed; and (4) when beset by problems and adversity, sustaining and bouncing back and even beyond (resiliency) to attain success. (p. 3)

This approach is based on the Conservation of Resources (COR) theory (Hobfoll, 2002), which posits that individuals seek to obtain, retain, and protect personal resources in order to control and impact their environment effectively. PsyCap is described as a positive interpretation of events that stimulates flourishing and success based on effort and constancy. According to Youssef-Morgan and Luthans (2013), the mechanisms through which PsyCap works focus on: (1) the intentionality and motivation for behavior; (2) positive cognitive assessments through which negative situations are reevaluated more positively; (3) positive emotions that facilitate the construction and restoration of weakened psychological resources, including the dimensions of PsyCap; and (4) social mechanisms that help in the development of personal resources. The concept extends to organizations and represents a competitive advantage because it is difficult to replicate. A study by Luthans et al. (2007b) shows that the four dimensions of PsyCap together are a better predictor of job performance and satisfaction than the four facets individually.

Improving PsyCap leads to greater organizational commitment, more favorable organizational citizenship behavior, less absenteeism, greater job satisfaction (Idris and Manganaro, 2017), and greater psychological well-being (Avey et al., 2011). Additionally, longitudinal studies show that PsyCap is a state-like construct, i.e., flexible and open to improvement (Avey et al., 2010; Peterson et al., 2011), and can be developed through short interventions (Luthans et al., 2006; Demerouti et al., 2011; Dello Russo and Stoykova, 2015; Ertosun et al., 2015). In order to carry out effective PsyCap interventions, it is important to take into account the organizational climate context because it seeks to promote positive thinking patterns. This transformation requires an organizational climate that promotes empowerment, support, and recognition (Luthans and Youssef-Morgan, 2017). PsyCap becomes relevant in the organizational context because high levels of its four dimensions make it possible to face adversities in organizational dynamics. Previous literature suggests that coaching offers the necessary conditions to cultivate this psychological resource (Petersen, 2015).

Whereas, research has focused on the impact of coaching on each of the dimensions separately, such as resilience (Grant, 2013a; Sherlock-Storey et al., 2013; Sarkar and Fletcher, 2016), hope (Green et al., 2006; Madden et al., 2011), and self-efficacy (Evers et al., 2006; Baron and Morin, 2009; McDowall and Butterworth, 2014), no workplace coaching studies have focused on the four dimensions of the PsyCap construct as a whole and their relationship with coaching (Hsu et al., 2019). Additionally, scientific evidence on the impact of PPMC on PsyCap is still missing, which is a new challenge and a novelty of this study. In addition, due to the lack of longitudinal studies that evaluate the maintenance of the results obtained in the coaching process over time (Grant and O’Connor, 2018), it is necessary to evaluate and verify the durability of the positive effects produced on PsyCap.

**Hypothesis 1**: Participants will increase their levels of PsyCap in Post time (after the intervention) for the Experimental group (EX) compared to Pre time (before the intervention), and compared to a Waiting List-control group (WL). Additionally, participants will report higher scores on PsyCap in Post time and 4 months after finishing the intervention (4-month follow-up; FUP) compared to Pre time (before the intervention), and considering the whole intervention group.

### Goal-Related Self-Efficacy and Goal Attainment in PPMC

Goals, as defined by Locke and Latham(2002, p. 705) are “the object or aim of an action, for example, to attain a specific standard of proficiency, usually within a specified time limit.” In other words, is the conscious intentionality that an individual does in order to achieve to desired results. Goal setting is the mechanism whereby the person reaches these goals. According to goal setting theory, difficult and specific goals lead to higher levels of performance as direct both attention and action (Locke and Latham, 2006). If the development of successful goals is perceived, individual’s confidence in their own capabilities enhances their ability to progress. Combined with self-efficacy, goal achievement leads individuals to set new, demanding goals (Schunk, 1990). This theory seems to fit properly in coaching literature because of the future-focused nature of goals and coaching, the key role of goal attainment in coaching, and the useful framework for coaching models provided by the goal setting theory, such as Specific, Measurable, Achievable, Realistic, Time-bound (SMART) (Clutterbuck and Spence, 2016).

By definition, coaching is a technique for learning and achieving goals by designing an action plan (Grant, 2013b). Goal attainment is an important indicator of the success of the process, according to the theory of coaching. The research finds coaching to be an effective method to achieve goals because it increases motivation, positive affect, and self-efficacy, and it facilitates goal progression (Grant and O’Connor, 2010; Grant, 2012). Specifically, strengths-based solutions reinforce individuals’ resilience skills and abilities and their use in achieving goals and making significant positive changes (Grant, 2011a). This perspective argues that coaches should spend most of the time posing inquiries that elicit the coachees’ thoughts about the best way to achieve their goals, rather than asking “why” questions that explore causality. By defining the different types of goals and their relevance in the clients’ transformation process, coaches can encourage their customers to gain insight and improve habits that enhance their job performance and, more importantly, their personal well-being and sense of self (Grant, 2019).

Bandura (1997) defined perceived self-efficacy as “beliefs in one’s capabilities to organize and execute the courses of action required to produce given attainments” (p. 3). Research suggests that people with higher levels of self-efficacy have stronger beliefs in their task-related capacities and their ability to set more ambitious goals and pursue them than people with lower levels of self-efficacy (Bandura, 1986). Coachee self-efficacy has been found to be a key antecedent of coaching outcomes, such as perceived coaching effectiveness (de Haan et al., 2013) and performance (Bozer et al., 2013). Considering the important role of behavioral and cognitive mechanisms in coaching, such as feedback, planning, and goal setting, and their connection to self-efficacy (Bandura, 1986), coachee self-efficacy is viewed as a central psychological factor in the process. Self-efficacy can be considered a generalized construct or a domain-specific variable to predict behavior and outcomes (Maddux, 2016). According to Bandura (1997) the more specific is self-efficacy, the greater prediction of successful behavior. We contend that the goal attainability construct can be better understood by taking into consideration the effects of goal-related self-efficacy in successfully fulfilling the tasks involved in the coaching process in order to reach goals. Evers et al. (2006) demonstrated that self-efficacy in setting goals has a positive impact on the client’s perceptions of coaching’s effectiveness. Given the relationship between these two concepts, specific self-efficacy for achieving goals will lead to greater progress in goal attainment.

Past research has proposed that workplace coaching has a positive impact on positive aspects, such as goal attainment (Grant, 2014), self-efficacy (Baron and Morin, 2009), and well-being (Theeboom et al., 2013). However, research on the effectiveness of a strengths-based micro-coaching intervention and its impact on these variables is still in its infancy (Peláez et al., 2020), and there is still a need for evidence-based research that considers specific self-efficacy as a predictor of goal attainment in PPMC. Moreover, there is a request in the scientific literature to relate goal-related self-efficacy and coaching outcomes (i.e., goal attainment; Bozer and Jones, 2018). In order to address this gap, we formulate the following hypothesis:

**Hypothesis 2**: Goal-related self-efficacy will predict goal attainment in the PPMC process.

## Materials and Methods

### Sample

The sample for this study was drawn from a multinational automotive industry company located in Spain, with 7,561 employees. Seventy-six employees who hold technical and engineering positions with non-supervisory or non-executive functions received an invitation to participate in a short-term strengths-based micro-coaching program. Finally, a total of 60 participants (79%) were involved in this research project: 35 participants divided into six groups that took part simultaneously and made up the experimental condition (EX group), and 25 participants divided into three groups that made up the waiting-list condition (WL group) as untreated comparisons in the study. Participants’ mean age was 36 years (*SD* = 7.5), and 70% were male. Furthermore, 82% of participants had a tenured contract, and the average length of time working in the company was 8.6 years (*SD* = 8.5). Participation was completely voluntary, and there was no extra financial incentive for their participation. All participants gave their written informed consent to release their personal data for scientific research purposes.

A degree of attrition was expected due to the longitudinal design of this study and the company’s casuistry. Due to unforeseen work-related and personal events, four employees did not complete the intervention program. Therefore, a total of 56 (93%) participants completed the program and responded to a post-intervention questionnaire, and 52 (87%) responded to the FUP questionnaire. For managerial reasons, the WL groups initiated the intervention shortly after the EX groups finished the coaching sessions (after the T2 evaluation), instead of waiting until the completion of the FUP questionnaires.

### Program Description and Procedure

The intervention was called the “Strengths-based micro-coaching program,” and it was designed for different purposes: (1) to present and provide feedback on the results of self-assessments of participants’ positive psychological resources (i.e., hope, optimism, resilience, and self-efficacy), well-being variables (i.e., work engagement), and healthy organizational outcomes (i.e., performance); and (2) to facilitate goal attainment by establishing an action plan based on the use of personal strengths.

In a previous study (Peláez et al., 2020), the authors explored the impact of this particular intervention program on work engagement and job performance. Thus, these two outcome variables were not included in the present study. This previous intervention program was extended over the course of 6 weeks and divided into a 2-hour group session and three individual coaching sessions. The intervention was delivered by four professional psychologists external to the organization with specific coaching and positive psychology expertise. They also participated in two group supervision sessions (one at the beginning and the second one in the middle of the process) with an experienced professional in this subject. All four coaches had to follow a guideline (i.e., protocol) in order to obtain uniform, and comparable information regarding the main issues on the coaching process. Moreover, each coach had to register the relevant points of the session based on the protocol. This procedure ensured that the results were based on the same approach.

The present study is related to the Peláez et al. (2020) study and has the same design and sample. We attempt to analyze the effectiveness of a PPMC program in increasing work-related variables (i.e., PsyCap), study the relationship between goal-related self-efficacy and goal attainment, and provide further evidence reinforcing its value and validity.

To manage this intervention, researchers were assisted by the manager of the plant in order to identify employees’ need to respond to high levels of job demands and reach higher performance goals. During the first phase of this project, employees were informed about the characteristics of the study, the evaluation procedure, the purpose of the intervention, and the confidentiality of their responses, according to the European data regulation standards. Furthermore, the research adhered to ethical principles and standards approved by the Research Ethics Committee of the University. Participants were not randomly allocated to either the EX group or the WL group because their assignment depended on their availability, the preferences of the organization, and coaches’ schedule. The participants could choose between the two groups through registering in a template sheet.

**FIGURE 1:**
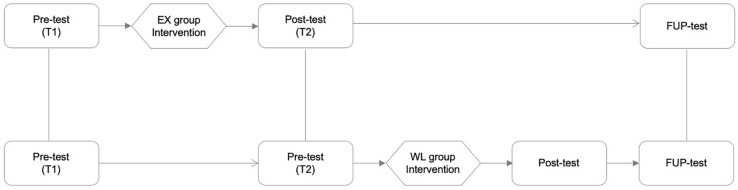
Experimental design of the study. EX: experimental group; WL: waiting list-control group; Pre-test: pre-assessment; Post-test: post-assessment; FUP-test: follow up-assessment; Tl: time 1; T2: time 2.

The study used a within-subjects (pre-post-FUP) and between-subjects (EX-WL) design. Participants were assessed at Time 1 (T1; before the intervention), Time 2 (T2; immediately after the intervention for the EX group, and before the intervention for the WL group), Post times (after the intervention for the whole intervention group, once the WL group has finished the intervention) and follow-up times (FUP; 4 months after finishing the intervention for the whole intervention group). The self-reported questionnaires were administrated online by sending an email with a direct link to each participant at all four assessment times. Next, participants in the experimental group started the 2-hour group session, followed by three micro-coaching sessions. Figure 1 represents the outline research of the study.

**FIGURE 2:**
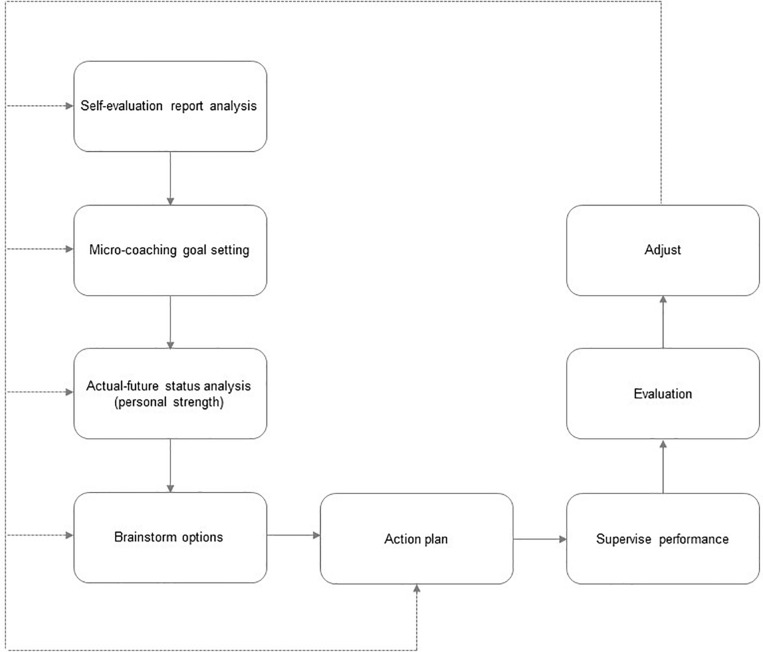
Intervention program model based on the RE-GROW model (Grant, 2011b).

The coaching sessions were grounded in Grant’s RE-GROW model (Review, Evaluate, Goal, Reality, Options, and Wrap up) (2011b) and the strengths-based approach (Linley and Harrington, 2006). Hence, the focus of the intervention was to set a specific goal for personal and professional growth, analyze the current-future status of the goal, brainstorm ways to achieve individual goals, establish an action plan, initiate action and implement the best options, supervise performance, evaluate progress between coaching sessions, and adjust actions if necessary (based on evaluation of progress). Following this approach, a self-regulatory cycle takes place that links outcomes from the previous session to the current session as the guiding thread in this micro-coaching process. Participants were guided by the coach through the different steps during the entire program. In addition, this model is expanded in the study with a previous step of a self-assessment report and analysis (see Figure 2).

During the group workshop session (i.e., the first session), participants received a short theoretical presentation on positive psychology, positive psychological coaching, and the variables assessed in the study. Next, the participants received an individual report and feedback on their self-assessment, providing a starting point and enhancing awareness of their personal resources, well-being, and performance. Following the structure, each coachee established a specific goal to focus on, and a working guide was offered that included a workbook, information, and instructions for coaching activities and a bibliography.

The program continued with 2 weekly 90-min individual micro-coaching sessions that mainly consisted of reporting the levels of goal-related self-efficacy, defining the goal and the action plan for achieving it. Throughout the intervention, the participants used their character strengths to reach the established goal. Specifically, in the “R” of the GROW model, the current status and personal strengths available to reach the desired status (goal) were identified, followed by a reflection on participants’ abilities, improvement areas, and external opportunities. Afterward, the individuals developed and initiated an action plan. Between sessions, participants worked on developing the plan. In each session, the coach helped the coachee to evaluate and adjust the goal or actions in order to obtain better results.

Finally, 2 weeks after finishing the two 90-min sessions, the participants attended a 60-min final follow-up session to monitor the action plan, celebrate the positive results and the accomplishment of the goal, and provide feedback on the program. To ensure transference of training back to their daily work, throughout this session, the “Best Possible Self” technique, developed by King (2001), was performed as a closing task, accompanied by visualization techniques based on their signature strengths. Participants were asked to picture themselves in the best possible future situation taking into account three specific areas (personal, professional, and social). Peters et al. (2010) found that this exercise was useful for improving personal and psychological well-being. In this intervention, this exercise was adapted to the individual coachee micro-coaching process and specific strengths used in the PPMC, encouraging participants to write down and then visualize the journey to achieve the goal using their personal resources. Table 1 summarizes the PPMC intervention program.

**TABLE 1:** Positive Psychological Micro-Coaching sessions framework.

Session	Main purpose	Activities/tasks	Homework
1	Connecting and sharing. Pre-assessment results: feedback and reflection. Goal setting. Workbook delivery.	Welcome: coaches’ presentation and objectives, structure and internal rules of the program. Ice-breaker: participants’ self-presentation through symbols. Positive Psychology inputs. Presentation of the variables assessed and delivery of the results. Goal setting using SMART + technique: role-playing in pair.	Brief survey to think about the gap between current and desired situation (i.e., How do you define success in your life at this moment? When are you at your best? What are your personal strengths?)
2	Process development following the GROW model: GOAL setting (SMART+), examine the REALITY, explore OPTIONS, and establish the WILL	Review session 1: potential areas uncovered (SMART + goal). **Reality:** identifying and reflecting about personal strengths and weaknesses (symbol identification, strengths map, SOWT analysis). **Options:** brainstorming, and analysis of advantages and disadvantages. **Action plan:** detailed description regarding the what, why, when, how, and who questions.	“Time line” exercise: steps to follow for the action plan. Start the action plan.
3	Follow-up the action plan:	Review session 2: contents and doubts. Activity: “Time line” adapted to the action plan. Reflection about the achievements so far and future actions. Activity: (written and visualized) “The Best Possible Self” exercise. Process overview	Practice and follow the plan.
4	Closing, review, and reflection	Review session 3: topics, action plan, and doubts. Coachees’ feedback: on the process, and coaches’ performance.	

**SMART, Specific, Measurable, Achievable, Realistic, Time-bound; +, Positive; SWOT, Strengths, Weaknesses, Opportunities, Threats.**

### Measures

#### Psychological Capital

Psychological capital was measured with the adapted version(Azanza et al., 2014) of the Psychological Capital Questionnaire (PCQ; Luthans et al., 2007a). The questionnaire consists of 12 items distributed in four factors: (1) self-efficacy (3 items; example item: “I feel confident in representing my work area in meetings with management.”); (2) hope (4 items; example item: “I can think of many ways to reach my current work goals.”); (3) optimism (2 items; example item: “I always look on the bright side of things regarding my job.”); and (4) resilience (3 items; example item: “I can get through difficult times at work because I’ve experienced difficulty before.”). The PCQ items were rated on a six-point Likert scale ranging from 1 (”strongly disagree”) to 6 (”strongly agree”). Based on the reliability test, PCQ obtained a coefficient of 0.809 for T1, 0.88 for T2, and 0.83 for T3 for the alpha Cronbach value, which means that this questionnaire can measure psychological capital consistently.

#### Goal-Related Self-Efficacy

Following Bandura’s (2006) guide for constructing self-efficacy scales, participants were asked during the first session to rate the degree of confidence to successfully achieve their goals, using a 10-point rating scale ranging from 0 (“cannot do”); through intermediate degrees of assurance, 5 (“moderately certain can do”); to complete assurance, 10 (“highly certain can do”). Although single-item measures are often avoided in research due to concerns about their psychometric properties, the challenge of applying research in practical contexts, such as the workplace has led to an examination of their suitability when circumstances require very brief scales that restrict the duration of the measurement design (Bowling, 2005). In this regard, previous research has demonstrated that a single-item self-reported measure of self-efficacy can be as effective as a multiple-item scale (Hoeppner et al., 2011; Williams and Smith, 2016). This advantage is important because a shorter survey is more likely to be answered by the participants (Nagy, 2002).

#### Goal Attainment

Participants established one goal that was related to the coaching program’s purpose and satisfied their specific needs. This variable was measured in the final session of the PPMC program to examine the coachees’ performance on the selected goal. As mentioned above, the use of a single-item scale in organizational research may be useful for capturing information if there are practical constraints (e. g., respondent load, reducing survey length) (Fisher et al., 2016). Based on this approach, goal attainment was assessed by asking the participants to rate their degree of success in attaining the goal through a percentage scale (example item: “What percentage of your goal have you achieved at this moment?”) from 0% (no attainment) to 100% (total attainment). Goal attainment scores were calculated by transforming percentages to scales from 1 to 10. This variable was measured in the 60-min final follow-up session.

### Data Analyses

Descriptive data analyses were calculated to test the relationships between the study variables using the SPSS 25.0 statistical program. In order to examine the effects of the intervention program, analysis of variance (ANOVA) with a 2 × 2 repeated measures design was conducted to analyze differences between-subjects factor (group: EX and WL) and within-subjects factor (time: T1; T2). While T1 refers to the first pre-intervention test for both EX and WL, T2 refers to the post-intervention test for EX and to the second pre-intervention test for WL, just before this second group started the intervention.

In addition, *t*-tests for related samples were performed to test for differences between Pre and Post times and Pre and FUP times considering the whole intervention group (EX and WL group), once the WL group had finished the intervention.

Moreover, following Cohen (1988), eta squared in the repeated-measures ANOVA and Cohen’s *d* as a measure of the effect size on *t*-tests for related samples were estimated (small effect = 0.01–0.03; moderate or intermediate effect = 0.03–0.05; large effect = 0.05). A significance level of 0.05 was established for all tests.

Finally, simple linear regression analyses were used to evaluate the specific link between the research variables (goal-related self-efficacy) at Pre time and the outcome variables (goal attainment) at Post time.

## Results

**FIGURE 3:**
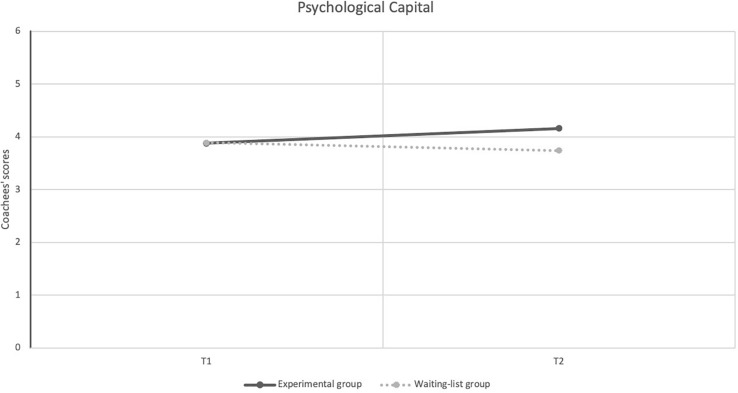
Dependent variable for each time factor (T1, T2) across groups.

First, 2 × 2 repeated measures (ANOVA) analysis was carried out, and results showed a statistically significant difference between the EX and WL groups on the dependent variable PsyCap [*F*(1.55) = 9.65, *p* < 0.05, η*_*p*_*^2^ = 0.152], demonstrating a large effect size. This result indicates that participants in the EX group had statistically higher levels of PsyCap at T2 (immediately after the intervention for EX, and before the intervention for WL) compared to T1 (Pre intervention time for both groups) and to WL. Figure 3 shows the interaction plots of the effects of the intervention program on PsyCap.

Next, paired-sample *t*-tests for the whole intervention group were performed to compare Pre and Post and Pre and FUP times (see Table 2). Results showed significantly higher levels of PsyCap at Post [*t*(53) = −5.22 *p* < 0.001, *d* = 1.42], and FUP [*t*(46) = −5.65 *p* < 0.001, *d* = 1.66] compared to Pre time, revealing large effect sizes. These findings suggest that the intervention had a positive impact on the development of the participants’ PsyCap, and that these effects remained high across time.

**TABLE 2:** Means and *t*-test on PsyCap for the whole group.

		*M*	*SD*	*t-*value	*df*	*p-*value
Pair 1	Pre	3.82	0.437	−5.22	53	0.000
	Post	4.12				
Pair 2	Pre	3.82	0.44	−5.65	46	0.000
	FUP	4.19				

**Pair 1, difference between Pre and Post time points for PsyCap; Pair 2, difference between Pre and FUP time points for PsyCap; M, mean; SD, Standard Deviation; df, degrees of freedom; p, significance level; Pre, pre-intervention time; Post, post-intervention time; FUP, follow-up time.**

Finally, in order to examine the relationship between goal-related self-efficacy and goal attainment, different analyses were performed. The average value of goal-related self-efficacy was 8.4 (*SD* = 1.3) with a minimum score reported of 5 and a maximum of 10 suggesting that the participants perceived medium-high levels of self-efficacy at the beginning of the process. For goal attainment the mean was 7.5 (*SD* = 4.9), the minimum 3 and the maximum 10 indicating that on average participants have reached 75% level of the established goal. Second, regression analyses were conducted to determine to what degree the independent variable (goal-related self-efficacy) contributes to the dependent variable (goal attainment). Results revealed that goal-related self-efficacy (*R*^2^ = 0.084, β = 0.29, *p* < 0.05) was a significant predictor of goal attainment in the short-term PPMC program; see Table 3.

**TABLE 3:** Regression analyses results for work-related self-efficacy as predictor of goal attainment.

Predictor	Adjusted *R*^2^	*B*	*SD*	β	*t*	*p*
Goal-related self-efficacy	0.084	0.53	0.25	0.29	2.15	0.037

**Dependent variable: goal attainment.**

## Discussion

The main aim of this study was to investigate the impact of a PPMC program on non-executive workers’ PsyCap and the connection between goal-related self-efficacy and goal attainment in the PPMC. Overall, the results agreed with this main objective of the study and confirmed the proposed hypotheses. Participants demonstrated significant increases in PsyCap after finishing the PPMC intervention and over time. Moreover, the results highlight the predictive role of goal-related self-efficacy in goal attainment in the coaching process. Therefore, results are consistent with previous research indicating that Positive Psychological Micro-Coaching (short-term and strengths-based) can be an effective and valuable intervention to enhance work-related outcomes and well-being, even when the number of coaching sessions is small (Theeboom et al., 2013). Finally, this study addresses a gap in the literature related to the few empirical control trial studies with a longitudinal design (Grant and O’Connor, 2018), in addition to investigating the relationship between goal-related self-efficacy and coaching outcomes (i.e., goal attainment) (Bozer and Jones, 2018).

The first hypothesis was supported in the current study. The results suggest that the intervention significantly increases PsyCap levels immediately after the intervention for the EX group when compared with the WL group. Findings also indicate a significantly increase in PsyCap after the intervention and 4 months after finishing it, compared to the baseline levels, considering the whole intervention group (once WL has finished the intervention). The state-like nature of PsyCap (Youssef-Morgan and Luthans, 2015) makes it suitable for interventions focused on personal growth (i.e., PPMC), and its working mechanisms (i.e., positive evaluation of the scenarios and opportunities to success based on effort and persistence; Youssef-Morgan and Luthans, 2013) confirm the positive and direct effect of PPMC. Based on the assumption that the coaching process pursues the capacity for growth of personal resources, these results are congruent with previous studies confirming that coaching provides the perfect environment for the development of PsyCap (Petersen, 2015). The effect of the non-executive PPMC program on PsyCap has not been previously investigated, and so these findings provide new scientific evidence in this regard.

The second hypothesis was also confirmed. The results revealed that goal-related self-efficacy is a significant predictor of goal attainment in the PPMC program; that is, participants’ goal-related self-efficacy enhanced positive outcomes (i.e., goal attainment) at the end of the intervention. Despite not having a baseline measurement for goal attainment (participants were asked only in the last coaching session about the level of achievement of the goal they established in the first session), an improvement in goal attainment was reported as stated by the participants during the last coaching session, and considering the high level of percentage achieved. Based on the RE-GROW model, interventions focused on achieving a specific goal and self-efficacy were shown to be a crucial precedent for coaching performance (de Haan et al., 2013). Because the coaching process aimed to help the coachee to set his/her own personal goals, it may have contributed to greater commitment to the goal and increased motivation to achieve it, followed by positive outcomes that are likely to strengthen feelings of self-efficacy (Bandura, 1997). As expected in this study, and in line with previous research (Evers et al., 2006), considering the essence of specific self-efficacy for achieving goals, the effect on goal attainment was positive and high. This finding addresses the gap in the literature and the request to relate goal-related self-efficacy to coaching outcomes (Bozer and Jones, 2018) and reinforce the importance of enhancing personal resources (i.e., self-efficacy) (Demerouti et al., 2011).

Moreover, results from this research contribute to the literature on coaching psychology by demonstrating that micro-coaching can be a useful positive intervention to improve optimal organizational functioning. Therefore, the study results are consistent with previous research showing that even if the number of coaching sessions is small, coaching can be successful (Theeboom et al., 2013; Peláez et al., 2020). The reason short-term coaching led to successful outcomes could be that the intervention focuses on developing specific skills and goals in a relatively brief period of time. Additionally, the findings strengthen the literature on empirical control trial studies with a longitudinal design, considering the effect of PPMC on work-related outcomes (i.e., PsyCap), long-term effects of coaching, and the role of self-efficacy in goal attainment.

### Implications for Practice

Some practical implications emerge from the study results. First, this study provides further evidence of the positive impact that PPMC has on employees’ personal resources and work outcomes, and it may contribute to the competitive advantage of an organization. In other words, investing in and developing employees’ personal resources is usually promoted in healthy organizations, understood as those that care about the psychosocial health of their workers (Salanova et al., 2012, 2019). This study has shown that relatively few coaching sessions can be effective, which could be an important element to consider given the challenges faced by organizations in turbulent and changing environments. People are working under time pressure and have to use their time effectively; under the paradigm of urgent vs. important, coaching may not be a priority task. In this regard, short coaching sessions are beneficial in terms of motivation, flexibility, costs, and parsimony, due to their focus on specific goals. In PPMC, not only positive resources are developed and reinforced, but also the coachee receive support in the development and use of techniques to handle challenging circumstances and cope with difficulties (Skinner and Zimmer-Gembeck, 2007). The complicated interactions between positive aspects of human functioning and negative experiences alter the way people think, feel and behave (Lomas and Ivtzan, 2016), and therefore should be taken into account in the coaching process.

Therefore, coaching provides opportunities not only to develop abilities and internalize them in everyday life (Evers et al., 2006), but also to increase the effectiveness of coachees’ functioning and work performance even when the environment is challenging. Workplace coaching needs to be agile, flexible, and easily integrated into the organization (Grant, 2016). Thus, workplace coaching, specifically PPMC, can serve as an important tool that can facilitate significant positive organizational change to address the problems that contemporary companies are experiencing. It is a short-term interaction designed to obtain long-term benefits.

### Limitations and Future Directions

Finally, some limitations of this study must be recognized. First, participants were not assigned randomly to either the EX or the WL group because the allocation depended on the participants’ availability and the organization’s priorities. Nevertheless, the findings of the *t*-test analysis between the groups did not show any significant difference in the outcome variable (PsyCap) at T1 (before the intervention).

Second, the sample was small and very specific; therefore, the result cannot be generalized. Therefore, future investigations should examine the effect of this intervention in other sectors or companies and extend the sample in order to contrast the results. Thus, replications are welcome in order to discover the benefits of the intervention based on its positive effects in other sectors, companies, or countries, and give greater validity to our findings.

Third, due to the organizational context, the comparison of the EX and WL groups at FUP was not possible because the WL group started the intervention shortly after the EX group finished it. Nonetheless, we found valuable results by comparing the whole intervention group across time (before, after, and FUP), calculating paired-sample *t*-tests. Future studies should consider adjusting the research design in order to compare the two conditions at this evaluation time. Additionally, we highlight the importance of a FUP evaluation to ensure the maintenance of the results over time and the use of objective or multisource ratings of outcome variables and the results.

Fourth, the self-efficacy and goal attainment measurements based on single-item scales are sensitive to bias and error. Additionally, the changes of self-efficacy and goal attainment were not possible to analyze since they were measured only once. Even so, our results were positive and congruent with previous research. However, the use of the Goal Attainment Scaling and the Self-efficacy Scale (Chen et al., 2001) should be considered in future studies for more accuracy, as well as evaluating the variables in different times in order to examine changes.

Fifth, our study is also limited by the use of self-reported data and thus it was not possible for the investigators to objectively determine the veracity of such data. Self-reported performance might boost social desirability (Caputo, 2017). Furthermore, as participation was voluntary, the competence and motivation of participants could have influenced our results. However, findings are consistent with the theory, and we attempted to minimize the impact of these biases in our study by collecting data over time (i.e., before, after and follow-up times). It could be valuable to include a wider range of objective measurements to examine the impact of this intervention. Also, it would be valuable to consider, not only the positive aspects of well-being, but also the evaluation of negative experiences and emotional states to gain a complete and realistic picture of well-being (Lomas and Ivtzan, 2016). Additionally, it could be interesting to assess in future studies the benefit and impact of PPMC on performance variables, such as behavioral persistence and performance flexibility (Theeboom et al., 2016).

Finally, even though positive and significant effects of PPMC were found on PsyCap and in the connection between goal-related self-efficacy and goal attainment, future research should consider focusing on specific factors in the effectiveness of coaching (e.g., performance, SMART goals, working alliance, commitment to the process) and on the analysis of the links between self-efficacy, goal attainment and changes on the outcome variable (PsyCap). Our study has shown that short-term coaching can be successful. However, a comparison of short-term and long-term interventions in future research would be very useful.

## Conclusion

To sum up, this study provides relevant information for both researchers and professionals. From a theoretical perspective, the results offer evidence about the effects of a Positive Psychological Micro-Coaching intervention on psychological capital and the predictability of goal-related self-efficacy on goal attainment during the coaching process. The present study presents original data indicating that short-term sessions are indeed effective in enhancing personal resources (i.e., PsyCap) and that on average participants reported medium-high percentage of attainment of their established goals. It also demonstrates that workplace coaching can increase PsyCap in non-executive workers, using a longitudinal controlled design. Although the effects of the intervention cannot be generalized, and comparisons of EX-WL at FUP were not possible, the encouraging results suggest that future studies should include stronger designs (i.e., multiple measurement points, and randomization). From an applied perspective, this research represents a significant development from an operational point of view because it provides professionals with an innovative and replicable intervention that can be adapted and implemented across a wide range of organizations. The findings highlight the strategic value of providing personal growth opportunities that can help employees to develop their skills to handle challenging circumstances and cope with difficulties, and therefore, contribute to successful organizational outcomes.

## Data Availability Statement

The raw data supporting the conclusions of this article will be made available by the authors, without undue reservation, to any qualified researcher.

## Ethics Statement

The studies involving human participants were reviewed and approved by the University Research Ethics Committee Universitat Jaume I. The patients/participants provided their written informed consent to participate in this study.

## Author Contributions

MP coordinated the entire intervention process, performed the data collection, and contributed to the wording of article. MS contributed during the intervention program and revised the manuscript. AC and MP developed the study design, conducted the analyses, and interpretation of the results. AC wrote the manuscript. All the authors listed have made a substantial intellectual contribution to the research and conceived the idea for the study.

## Conflict of Interest

The authors declare that the research was conducted in the absence of any commercial or financial relationships that could be construed as a potential conflict of interest.

## Abbreviations

PPMC: positive psychological micro-coaching
PsyCap: psychological capital
PRE: pre-assessment time
POST: post-assessment time
FUP: follow up time
EX: experimental group
WL: waiting-list control group
ANOVA: Analyses of Variance.
