# Corrigendum: Positive Psychology Micro-Coaching Intervention: Effects on Psychological Capital and Goal-Related Self-Efficacy

**DOI:** 10.3389/fpsyg.2021.669283

**Published:** 2021-03-17

**Authors:** Alina Corbu, María Josefina Peláez Zuberbühler, Marisa Salanova

**Affiliations:** WANT Research Team, Department of Social Psychology, Universitat Jaume I, Castellón de la Plana, Spain

**Keywords:** positive psychology coaching, goal-related self-efficacy, psychological capital, goal attainment, short-term coaching, control trial, strengths-based intervention

In the original article, the reference for Castiello D'Antonio (2018) was incorrectly written as Castiello, D., and Antonio, A. (2018). Coaching psychology and positive psychology in work and organizational psychology. *Psychol. Manage. J*. 21, 130–150. doi: 10.1037/mgr0000070

It should be Castiello D'Antonio, A. (2018). Coaching psychology and positive psychology in work and organizational psychology. *Psychol. Manage. J*. 21, 130–150. doi: 10.1037/mgr0000070

The authors apologize for this error and state that this does not change the scientific conclusions of the article in any way. The original article has been updated.
